# COMMUNITY-ENGAGED RESEARCH WITH SOCIAL SERVICE ORGANIZATIONS FOR OLDER ADULT MENTAL HEALTH EQUITY

**DOI:** 10.1093/geroni/igae098.0458

**Published:** 2024-12-31

**Authors:** Lesley Steinman, Mary Mitchell, Sherry Wu

**Affiliations:** Chair; Co-Chair; Discussant

## Abstract

Twenty years ago, our research center collaborated with local community-based social service organizations to co-create and evaluate PEARLS, a community-based, collaborative care model for late-life depression. PEARLS builds capacity among trusted, front-line, non-clinical providers (e.g., case managers, community health workers) to be depression care managers, taking a “task shifting” and “task sharing” approach recommended to close the mental health treatment gap by improving access to quality care where older adults live, work, play, pray, and age. While PEARLS was developed with, in, and for communities underserved by clinical mental health care, for the past five years our community-academic partnership has focused on reaching older adults who have been historically marginalized by racism, sexism, ageism, and other structural inequities. The five-year PEARLS Equity Study is supported by CDC Prevention Research Center funding to improve older adult mental health equity through active community collaborations from design to dissemination. This symposium will bring feature several of our local PEARLS partners to highlight the value of community-engaged dissemination and implementation research to fortify the feasibility, equity, and sustainability of studies to improve the lives of older adults through the social service organizations working to support aging in place and quality of life. We will provide real-world examples that embody the GSA conference theme, the Fortitude Factor, offering take-aways for researchers, practitioners, and policymakers to strengthen how community-academic partnerships are essential for generating and integrating research findings into aging practice and policy.

